# A patient with recurrent syncope—it does matter how slow and long you go

**DOI:** 10.1007/s12471-023-01779-y

**Published:** 2023-05-03

**Authors:** Eva Roseboom, Alexander H. Maass, Jozine M. ter Maaten

**Affiliations:** Department of Cardiology, University Medical Centre Groningen, University of Groningen, Groningen, The Netherlands

## Answer

**Fig. 1:**
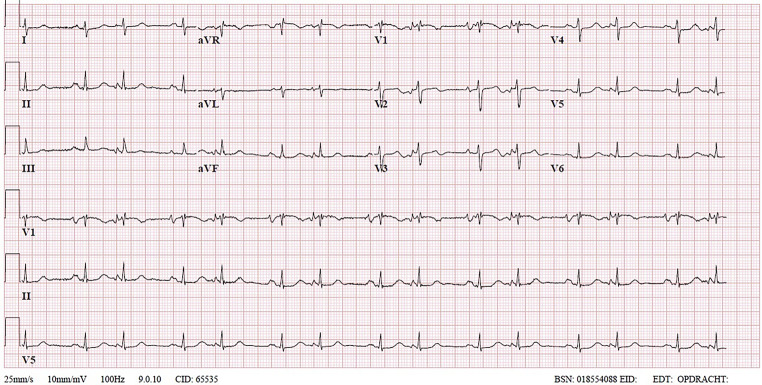
Electrocardiogram after cessation of sotalol showing restoration of conduction intervals and normal conduction of premature atrial contractions

Torsade de pointes is a polymorphic ventricular tachycardia (VT) with cyclic alteration of the QRS axis, in the context of prolonged repolarisation [1]. Stereotypically, it is initiated by an early afterdepolarisation in a short-long-short sequence. Fig. 1b (in the question) shows VT with characteristic sinusoidal alteration of the QRS axis, which is only preceded by a single long-coupled premature ventricular complex arising from the T wave in prolonged QT interval.

Our patient developed long QT syndrome due to the use of sotalol. Current guidelines on atrial fibrillation suggest sotalol may be considered for long-term rhythm control [2]. Sotalol is a nonselective beta-blocker with class III antiarrhythmic properties, blocking potassium efflux and prolonging phase 3 of the action potential. It acts in a dose-dependent manner and exhibits reverse use dependence, being more potent in bradycardia. Therefore, an ambulatory 24-hour ECG recording is recommended to evaluate QT interval prolongation and bradyarrhythmias. Since sotalol is renally excreted, it is vital to monitor kidney function throughout treatment and implement dose adjustments accordingly. Proarrhythmia is reported in 1–4% of sotalol users, with a higher prevalence in women and patients with serum creatinine > 124 μmol/l [3].

Similar to all beta-blockers, sotalol can lead to conduction slowing. The appearance of fragmentation of T waves in Fig. 1a (in the question) are in fact blocked premature atrial contractions (PACs) in bigeminy. An ECG recorded after cessation of sotalol showed restoration of conduction intervals and normal conduction of PACs (Fig. 1). The patient was discharged with bisoprolol and scheduled for cavotricuspid isthmus ablation.

## References

[1] Khan IA (2002). Long QT syndrome: diagnosis and management. Am Heart J.

[2] Hindricks G, Potpara T, Dagres N, Arbelo E, Bax JJ, Blomström-Lundqvist C (2021). ESC Scientific Document Group. 2020 ESC Guidelines for the diagnosis and management of atrial fibrillation developed in collaboration with the European Association for Cardio-Thoracic Surgery (EACTS): The Task Force for the diagnosis and management of atrial fibrillation of the European Society of Cardiology (ESC) Developed with the special contribution of the European Heart Rhythm Association (EHRA) of the ESC. Eur Heart J.

[3] Lehmann MH, Hardy S, Archibald D, Quart B, MacNeil DJ (1996). Sex difference in risk of torsade de pointes with d,l-sotalol. Circulation.

